# Trends of physical activity and recreational screen time among Chinese children and adolescents: a national study from 2017 to 2019

**DOI:** 10.1186/s12889-024-18822-1

**Published:** 2024-05-13

**Authors:** Ming Ming Guo, Koon Teck Koh, Xiao Zan Wang

**Affiliations:** 1 College of Physical Education and Health, East China Normal University, Shanghai, China; 2 Physical Education & Sports Science, National Institute of Education, Nanyang Technological University, Singapore, Singapore

**Keywords:** Chinese children and adolescents, National trends, Physical activity, Recreational screen time

## Abstract

**Background:**

The prevalence of physical inactivity and sedentary behavior among children and adolescents is a growing public health concern. This study aims to examine the trends in Physical Activity (PA) and Recreational Screen Time (RST) amongst children and adolescents in China, considering variations in genders, school levels, areas (urban versus rural), and regions (north versus south). The findings provide a foundation to guide policy and strategy making for future health promotion and development.

**Methods:**

An annual national cross-sectional survey was conducted in China from 2017 to 2019 cumulatively involving 52,503 (48% female) children and adolescents from grades 4 to 12 (aged 12.72 ± 2.12). Data on PA and RST were collected through self-administered questionnaires. Weighted least squares regression was used to analyze the trends and differences in PA and RST among the participants’ profiles.

**Results:**

There was an annual decreased in PA compliance rate of approximately 3.43% (95% CI: 0.79-6.08%) for primary school students, primarily among males residing in rural areas, and in northern regions. Middle school students experienced a yearly decrease of about 5.23% (95% CI: 2.55-7.92%) in PA compliance across all genders, regions, and urban areas. Similarly, the RST compliance rates for primary school students declined by approximately 3.18% (95% CI: 1.57-4.78%) annually for all genders and areas, but only in the northern regions.

**Conclusions:**

This research highlights a downward trend in PA and RST compliance amongst Chinese children and adolescents, with variations based on school level, gender, area, and region. Urgent policies and interventions are imperative to promote PA while mitigating excessive RST within these populations.

**Supplementary Information:**

The online version contains supplementary material available at 10.1186/s12889-024-18822-1.

## Introduction

Physical activity (PA) refers to any bodily movement that causes energy expenditure in skeletal muscles [1]. Recreational Screen Time (RST) includes all discretionary screen time (e.g., nonemployment or non-school related) taken while sedentary, typically encompassing activities such as television viewing, video gaming, and computer use [2]. Previous studies have demonstrated that insufficient PA and excessive RST can significantly affect the physiological and psychological health of children and adolescents, thereby affecting their quality of life and daily performance. These health impacts include an increased incidence of myopia, obesity, type 2 diabetes, anxiety, depression, and cardiovascular diseases [3]. Additionally, these conditions are associated with reduced sleep quality, diminished levels of physical fitness, and impaired cognitive abilities and academic performance [3]. Children and adolescents who fail to achieve 60 min of Moderate-to-Vigorous Physical Activity (MVPA) per day as advised by the World Health Organization’s (WHO) guidelines are considered physically inactive [3]. Furthermore, Canada’s 24-hour movement guidelines suggest that children and adolescents should restrict their RST to under 2 h per day [2]. Despite these guidelines, studies have shown that worldwide compliance rates amongst children and adolescents are low – only 19% of children and adolescents meet WHO’s PA recommendations, while a mere 34–39% comply with Canada’s RST guidelines [4, 5]. Given the alarming levels of PA and RST amongst children and adolescents worldwide, the WHO aims decrease global physical inactivity by 15% by 2030 [3]. 

Compared to other countries around the world, Chinese children and adolescents have lower levels of PA and longer RST. According to the Global Report Card on Physical Activity for Children and Youth, China is one of the lowest ranked countries in terms of PA and RST indicators [6]. This has led to an increase in obesity rates among Chinese children and adolescents in recent years, as well as healthcare expenditures [7]. More importantly, if this trend persists, it is likely to cause a decline in China’s future human resources [8]. Cui and Zhang suggested that the low levels of PA and high RST amongst Chinese children and adolescents may be associated with China’s economic development and the rapid proliferation of electronic devices. Specifically, China’s rapid economic growth has reduced the likelihood of children and adolescents assisting with agricultural or industrial production in their families, altered their modes of commuting, thereby decreasing their levels of PA. Furthermore, the wide spread of electronic devices has attracted significant number of the children and adolescents to spend substantial amounts of time on smartphones or tablets. This not only increases their RST but also further diminishes the possibility of them engaging in outdoor PA [9, 10]. This was especially so in the early 21st century, where China’s economic growth surpassed the annual Gross Domestic Product (GDP) growth rate of 9%, and the prevalence of smartphone usage amongst children and adolescents increased by more than 10% annually [11, 12]. However, there is a lack of research examining the current trends in PA and RST amongst Chinese children and adolescents, which presents a gap in the literature.

Previous studies have pointed out that there are significant disparities in children and adolescents’ PA levels based on their school levels, areas (urban versus rural), and regions. Male students also tend to display more PA and RST than females, primarily due to societal cultural factors and gender role expectations that restrict opportunities and reduce social support for PA among females, and also because males are more predisposed towards screen-based entertainment activities, such as video games [3]. Additionally, changes in academic pressure and living conditions result in lower school-level students engaging in more PA and less RST than their higher school-level counterparts [13]. Children and adolescents residing in urban regions also display more PA and less RST compared to those living in rural areas because there are more opportunities and accessibilities for PA [9]. Regional factors such as favorable temperature, better air quality, and higher level of economic development also provide a conducive environment for children and adolescents to participate in PA [14–16]. However, findings from these cross-sectional studies are unable to adequately predict current trends in PA and RST, and hence, have limited contribution to guiding policy formulation and designing health promotion programs.

Previous literature has explored the changing trends in PA and RST amongst children and adolescents. On one hand, studies by Ng et al. and Knuth et al. found due to lifestyle changes, PA amongst children and adolescents over the last few decades have decreased significantly [17, 18]. Moreover, research conducted in the United States and China showed a global increasing trend in RST amongst children and adolescents due to widespread screen consumption [19, 20]. On the other hand, Guthold et al. and Kalman et al. argued that the PA of children and adolescents worldwide is slowly increasing due to the implementation of various health promotion interventions in different countries [4, 21]. For instance, based on the reports by the Active Healthy Kids Global Alliance, there appears to be a decreasing trend in RST amongst children and adolescents globally over the past decade. A reason for this could be due to changes in the rating method adopted by such reports. In 2018, these reports subdivided the D grade into D-, D, and D+ (representing 20-26%, 27-33%, and 34–39% of children and adolescents meeting the RST guidelines, respectively), which resulted in the global percentage rating of children and adolescents meeting the RST guidelines to subsequently shift from D to D+ [5, 6, 22]. Consequently, it may not be meaningful to compare trends in RST amongst children and adolescents solely based on grades.

These conflicting results, combined with the lack of previous research investigating the changing trends in PA and RST amongst children and adolescents of different genders, school levels, areas, and regions thereby limit our understanding. This is especially so for highly digitalized countries with rapid economic development such as China. To close this gap, a large-scale cross-sectional survey over 3 consecutive years (2017–2019) was conducted. Specifically, two research questions guide the present study: (1) what are the changing trends in PA and RST amongst children and adolescents in China? And (2) how do these trends differ between genders, school levels, areas (urban versus rural), and regions (north versus south)?

## Materials and methods

### Study design and participants

This research was conducted as part of the “Construction of a Big Data Platform for Children and Adolescents’ Sports and Fitness in China (CBDPCASF)” project. The CBDPCASF project, funded by the National Social Science Foundation of China (Grant No. 16ZDA228), aims to establish a comprehensive platform for collating health and fitness-related data for Chinese children and adolescents. Between 2017 and 2019, the research team sent research invitations to schools involved in the project from September to December annually. Schools that accepted the invitation randomly selected students (two classes in each grade) from 4 to 12 grades (aged 10 to 18 years old) to participate in the study. Students below grade 4 were not invited to participate in the study because they might not understand the questionnaire content well, potentially leading to invalid responses.

During the period from 2017 to 2019, a total of 52,503 Chinese children and adolescents from 150 schools across 45 cities ranging from grades 4 to 12 participated in this study. The average age of these participants was 12.72 ± 2.12 years, and among these participants, 25,322 were female, accounting for 48% of the total. The annual number of participants from 2017 to 2019 was 22,820, 16,061, and 13,622 respectively.

### Procedures

Informed consent from participants and their guardians were obtained before initiating the survey each year. Subsequently, participants received a web link to a questionnaire from their teachers, which was hosted on the Questionnaire Star platform [23]. The questionnaire comprised a total of 20 questions, including participants’ demographic details (school names, grades, genders), and their PA and RST for the previous week. The survey required approximately 15 min to complete. Participants were asked to complete the questionnaire either in the computer classroom in school or at home using their parents’ mobile phones. For geographic and regional analysis of PA and RST, the areas (urban/rural) and regions (north/south) were determined based on the name of the school that the participants attended, using the Baidu Map Open Platform [24]. Privacy protection protocols were adhered to by de-identifying or removing all personal identifiers, thereby ensuring the confidentiality and rigor of this study. Data regarding the corresponding Chinese population, categorized by year, gender, school level, area, and region were procured from the China Statistical Yearbook to calculate weighted average compliance rates [11, 25, 26]. Finally, participants with incomplete information, incorrect responses, abnormal results, outliers (based on Tukey’s method) [27], and those unable to engage in PA due to exceptional circumstances or pre-existing medical conditions (such as disabilities or fractures) during the survey period were excluded.

### Instruments

The Chinese adaptation of the Physical Activity Questionnaire for Older Children (PAQ-C) was employed to evaluate the participants’ PA [28]. The PAQ-C is a self-report tool used to assess PA level based on a 7-day recall period. Its reliability and validity have been established in previous studies, with a Cronbach’s alpha coefficient of 0.821, demonstrating its suitability for assessing PA amongst Chinese children and adolescents [29, 30]. The PAQ-C comprises of 10 items, starting with a question on the frequency of engagement in various PA such as swimming, martial arts, and basketball. Items 2 to 8 focus on the PA status during specific intervals, such as during physical education classes, recess, after school, evenings, and weekends. The penultimate item asks about the frequency of engaging in PA for more than thirty minutes daily in the past week. The final item focuses on special circumstances that might have impeded PA in the past week. The first 9 questions employ a five-point Likert scale, with 1 indicating the lowest level or frequency of PA participation, while 5 represents the highest level or frequency of PA participation. The final question offers 2 options, “Yes” and “No”, for participants to indicate whether they were restricted from engaging in PA in the past week due to any special circumstances. The final PA score was s calculated as the average of scores from questions 1 to 9, reflecting the participants’ level of PA over the past 7 days [28]. Furthermore, based on the comparative study of PAQ-C and accelerometer data by Voss et al., a PAQ-C score above 2.87 essentially equates to a daily MVPA time of more than 60 min. Therefore, the present study considered a PAQ-C score above 2.87 to be in compliance with the PA guidelines [29]. 

Participants’ RST was assessed using the Chinese adaptation of the Adolescent Sedentary Activity Questionnaire (ASAQ) [31]. This self-administered instrument uses a 7-day recall period to assess children and adolescents’ sedentary time. Its reliability and validity in studying sedentary time amongst Chinese children and adolescents have been previously established by several studies, with a Cronbach’s alpha coefficient of 0.729 [31, 32]. The ASAQ contains 6 items that investigate participants’ RST over the past 7 days. These items encompass the daily time spent watching TV or movies, and using smartphones, computers, or tablets (excluding time spent for study and work purposes). The calculation of participants’ weekly RST is achieved by the total sum of the 6 items. The average daily RST is obtained by dividing the weekly RST by 7 [31]. According to the Canadian 24-hour Movement Guidelines and the Chinese Children and Adolescents’ Physical Activity Guidelines [2, 33], participants with less than 2 h of daily RST are considered to have met the RST guidelines.

Participants’ area (urban/rural) was determined using the geographical location of their schools and the Application Programming Interface (API) provided by Baidu Maps’ open platform [24]. This platform is created by Baidu, China’s largest map service provider. It offers services like maps, location search, route planning, and positioning. This platform allows users to extract information such as administrative regions and urban-rural classifications for specific locations published by the Chinese government [24]. Given China’s policy that requires students to attend schools near their residence, the school’s location is therefore considered representative of the student’s geographical location [34]. Based on the names of the schools that participants attended, we were able to obtain their provinces, cities, and areas (rural versus urban) classification using the Baidu Maps Open Platform. Based on participants’ provinces and cities information, we categorized their regions as either “North” or “South”. Participants located north of the Qinling-Huaihe Line were classified under the “North” region, while those located south of the Qinling-Huaihe Line was classified under the “South” region. The Qinling-Huaihe Line was used as it serves as a natural geographical dividing line in China. The south region of this line generally experience higher temperatures, humidity, and better air quality as compared to those in the north region [35]. 

The total population data for each participant group was extracted from the Chinese Statistical Yearbook [11, 25, 26] and was subsequently used as weighting information during data analysis. The Chinese Statistical Yearbook is an annual publication that comprehensively reflects the economic and social development of China. It systematically includes data – the number of students in each region, province, area, school level, and gender – at the national level, as well as across various provinces in China [11]. 

### Statistical analyses

All samples were grouped based on the year, region, province, area, school level, and gender categories. The PA and RST compliance rates for each group were computed. To enhance the representativeness of the data, Mixed Linear Regression (MLR) [4] was used to estimate the missing rural or urban compliance rates for provinces that included only urban or rural participants. This estimation was carried out by leveraging on data from provinces with both urban and rural participation. The total population of each group previously obtained from the Chinese Statistical Yearbook was incorporated into the dataset as a weighting variable for subsequent analyses [20]. 

Following this, the weighted average PA and RST compliance rates and the 95% Confidence Intervals (95% CI) were calculated based on participants’ region, area, school level, and gender. The Weighted Least Squares Regression (WLS) was used to evaluate linear trends in PA and RST compliance rates from 2017 to 2019. Absolute differences in the PA and RST compliance rates by year, region, area, school level, and gender were also determined using WLS based on the data collected during this period (i.e., from 2017 to 2019). For ease of data visualization, the trends of PA and RST compliance amongst children and adolescents of different regions, areas, school levels, and genders were presented using line graphs. All data processing and analyses were conducted using Python (Python 3.10, Python Software Foundation, Wilmington, DE, USA) with the statsmodels, scipy, seaborn, and matplotlib extension libraries [36]. A two-sided significance level was set at 0.05.

## Results

### Participant characteristics

Following data cleaning processes, a total of 11,353 participants (21.62%) were excluded from the analysis. The exclusion was due to several reasons: 24 participants (0.05%) lacked the necessary grade information; 3,147 participants (5.99%) could not participate in PA during the survey period due to various circumstances, such as illness or menstrual cycles for female participants; 4,746 participants (9.04%) submitted incorrect responses; 1,491 participants (2.84%) reported total sitting times that exceeded 24 h per day; 80 participants (0.15%) had a sample size smaller than 10 after being grouped by year, province, school level, gender, area, and region; 1,865 (3.55%) of participants’ PA scores or overall sitting time were considered as outliers. As a result, the final analysis was conducted using a dataset of 41,150 individuals, including 20,262 females (49%). This sample comprised of 22,143 primary school students, 13,320 middle school students, and 5,687 high school students. Notably, the annual sample size varied: for primary school students, it ranged from 4,458 to 12,282; for middle school students, it was between 2,973 and 5,484; and for high school students, it fell between 1,755 and 2,053. Unweighted and weighted sample sizes for each year, school level, gender, area, and region are respectively provided in Table 1 and sTable 1.

### Physical activity

Results from the present study showed that in 2019, only a handful of children and adolescents met the recommended PA level set by WHO – 25.33% (95% CI: 21.05-29.61%) of primary school students, 17.61% (95% CI:14.68-20.55%) of middle school students, and 12.56% (95% CI: 4.37-20.75%) of high school students met the guidelines (Table 2).

**Fig. 1:**
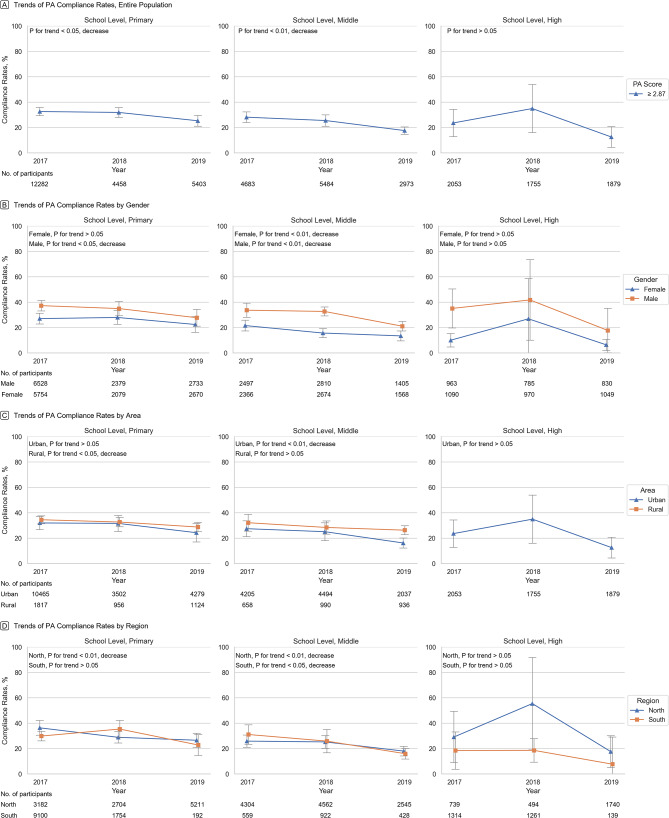
Crude weighted trends in physical activity from 20,017 − 2019. Data were weighted to be nationally representative. Error bars indicate 95% CIs

From 2017 to 2019, the average annual decrease in PA compliance rates was 3.43% (95% CI: 0.79-6.08%) for primary school students, 5.23% (95% CI: 2.55-7.92%) for middle school students, and 5.35% (95% CI: 2.67-8.03%) for male students. For rural and urban students, the average annual decrease in PA compliance rates was 2.89% (95% CI: 1.03-4.75%) and 4.37% (95% CI: 1.43-7.31%), respectively. For students in the northern and southern regions, the average annual decrease in PA compliance rate was 4.82% (95% CI: 1.97-7.66%) and 3.6% (95% CI: 0.37-6.84%), respectively. However, PA compliance rates remained constant during this period for high school students, and for female students. The downward trend in PA compliance amongst primary school students was primarily driven by a reduction in PA compliance amongst male students, students in rural areas, and students in northern regions. The decline in PA compliance rates amongst middle school students was observed across all genders, regions, and urban areas, but was not among rural areas. Analysis of compliance rate trends for PA by genders, areas, and regions, categorized according to school levels can be found in sTable 2. For overall prevalence estimates, please refer to Table 2; Fig. 1.

**Table 1:** Sample size for physical activity and recreational screen time in Chinese children and adolescents from 2017–2019 ^a^

	No. of Participants by School Level (Weight%)^b^								
	2017			2018			2019		
	Primary	Middle	High	Primary	Middle	High	Primary	Middle	High
**Overall**	12,282 (100)	4863 (100)	2053 (100)	4458 (100)	5484 (100)	1755 (100)	5403 (100)	2973 (100)	1879 (100)
**Gender**									
Male	6528 (54)	2497 (54)	963 (54)	2379 (54)	2810 (54)	785 (54)	2733 (54)	1405 (54)	830 (54)
Female	5754 (46)	2366 (46)	1090 (46)	2079 (46)	2674 (46)	970 (46)	2670 (46)	1568 (46)	1049 (46)
**Area** ^**c**^									
Urban	10,465 (73)	4205 (86)	2053 (100)	3502 (74)	4494 (86)	1755 (100)	4279 (76)	2037 (87)	1879 (100)
Rural	1817 (27)	658 (14)	0 (0)^e^	956 (26)	990 (14)	0 (0) ^e^	1124 (24)	936 (13)	0 (0) ^e^
**Region** ^**d**^									
North	3182 (41)	4304 (41)	739 (43)	2704 (40)	4562 (41)	494 (43)	5211 (40)	2545 (41)	1740 (43)
South	9100 (59)	559 (59)	1314 (57)	1754 (60)	922 (59)	1261 (57)	192 (60)	428 (59)	139 (57)

^a^ Participant characteristics are presented by year and school level, namely: primary school, middle school, and high school students. The sample size was weighted to represent national demographics, with the primary school group constituting 59.69%, the middle school group making up 26.27%, and the high school group comprising 14.04% in 2017. In 2018, the distributions were 59.53% for the primary school group, 26.79% for the middle school group, and 13.68% for the high school group. In 2019, the respective proportions were 59.32% for the primary school group, 27.11% for the middle school group, and 13.56% for the high school group, collectively representing the population of Chinese school students.

^b^ Please note that the weighted percentages may not add up to 100% due to the presence of missing data.

^c^ Area classifications were determined based on the urban-rural designation of each school, as provided by the Baidu Maps Open Platform.

^d^ Regions were defined according to the natural geographic boundaries of China, specifically the Qinling-Huaihe Line.

^e^ Given the prevalence of high schools in urban areas across China, the value in this particular context is registered as 0.

**Table 2:** Crude weighted trends in physical activity and recreational screen time compliance rates among Chinese children and adolescents from 2017–2019 ^a^

	Trends in Physical Activity and Recreational Screen Time Compliance Rates					
	2017	2018	2019	β (95% CI) ^b^	*P* for Trend ^b^	2019 VS 2017 Difference (95% CI) ^c^
**Physical Activity Compliance Rates, Weighted % (95% CI)**						
School Level						
Primary	32.61 (29.36, 35.86)	31.89 (27.98, 35.8)	25.33 (21.05, 29.61)	-3.43 (-6.08, -0.79)	0.011	-7.28 (-12.41, 3.35)
Middle	28.15 (23.97, 32.33)	25.53 (21.07, 29.98)	17.61 (14.68, 20.55)	-5.23 (-7.92, -2.55)	< 0.01	-10.54 (-14.45, 0.7)
High	23.59 (12.83, 34.36)	34.97 (16.01, 53.93)	12.56 (4.37, 20.75)	-4.09 (-14.98, 6.79)	0.448	-11.03 (-27.21, 10.39)
Gender						
Male	36.3 (33.2, 39.4)	35.01 (30.66, 39.36)	25.11 (21.58, 28.63)	-5.35 (-8.03, -2.67)	< 0.01	-11.19 (-16.07, -2.44)
Female	24.44 (21.27, 27.6)	24.86 (20.09, 29.63)	18.69 (15.08, 22.31)	-2.69 (-5.42, 0.04)	0.053	-5.74 (-12.31, 2.81)
Area						
Rural	34.12 (31.45, 36.8)	31.95 (29.09, 34.82)	28.23 (26.03, 30.44)	-2.89 (-4.75, -1.03)	0.003	-5.89 (-9.99, 2.07)
Urban	30.04 (26.44, 33.63)	30.24 (25.43, 35.05)	20.78 (17.18, 24.38)	-4.37 (-7.31, -1.43)	0.004	-9.26 (-17.04, -1.59)
Region						
North	32.84 (28.72, 36.95)	29.99 (25.13, 34.84)	23.27 (19.98, 26.55)	-4.82 (-7.66, -1.97)	0.001	-9.57 (-14.3, 0.85)
South	29.2 (25.96, 32.44)	31.21 (26.33, 36.09)	19.69 (15.65, 23.73)	-3.6 (-6.84, -0.37)	0.029	-9.51 (-17.74, -0.75)
**Recreational Screen Time Compliance Rates, Weighted % (95% CI)**						
School Level						
Primary	86.47 (84.79, 88.15)	80.45 (77.86, 83.03)	80.55 (77.96, 83.14)	-3.18 (-4.78, -1.57)	< 0.01	-5.92 (-7.45, 0.02)
Middle	87.14 (85.18, 89.09)	82.92 (78.67, 87.17)	82.72 (79.12, 86.32)	-2.23 (-4.59, 0.12)	0.063	-4.41 (-5.86, 3.46)
High	92.14 (90.05, 94.23)	90.15 (85.29, 95.01)	90.52 (85.43, 95.6)	-0.91 (-3.68, 1.86)	0.508	-1.62 (-11.28, 6.0)
Gender						
Male	85.08 (83.48, 86.67)	78.73 (75.63, 81.83)	80.71 (77.64, 83.78)	-2.42 (-4.32, -0.53)	0.013	-4.37 (-6.16, 3.03)
Female	89.47 (87.9, 91.03)	86.15 (84.35, 87.96)	83.33 (80.91, 85.76)	-3.08 (-4.39, -1.77)	< 0.01	-6.13 (-6.5, 1.48)
Area						
Rural	83.56 (82.5, 84.62)	80.51 (78.32, 82.69)	79.26 (77.46, 81.06)	-2.21 (-3.38, -1.04)	< 0.01	-4.3 (-4.2, 1.72)
Urban	87.99 (86.36, 89.62)	82.32 (79.45, 85.19)	82.52 (79.74, 85.3)	-2.89 (-4.62, -1.16)	0.001	-5.47 (-7.17, 1.95)
Region						
North	89.58 (88.14, 91.03)	82.11 (79.83, 84.39)	81.15 (78.68, 83.61)	-4.17 (-5.71, -2.63)	< 0.01	-8.43 (-8.94, -1.12)
South	84.97 (83.36, 86.59)	81.85 (78.19, 85.51)	83.64 (80.57, 86.71)	-1.08 (-3.16, 0.99)	0.301	-1.33 (-3.85, 5.81)

^a^ Weighted estimates and 95% confidence intervals (CIs) were calculated for each survey year. These estimates were adjusted to provide nationally representative data.

^b^ The parameter estimates β, corresponding 95% CIs, and *P* values for trend were obtained using linear regression, treating the year cycle as a continuous variable. β can be interpreted as the average percentage point change in prevalence per annum.

^c^ A value below zero indicates a decrease, corresponding to a negative difference.

Upon aggregating data from 2017 to 2019, we found that during the primary school stage, PA compliance consistently decreased with each increase in grade level (Odds Ratio [OR] = 0.98, 95% CI:0.97–0.99 for each grade increment compared to the previous grade). However, during the high school, PA compliance increased consistently with each increase grade level (OR = 1.13, 95% CI:1.11–1.14 for each grade increment compared to the previous grade). We also found that across all school levels, the PA compliance of females was significantly lower than that of males, and this gender gap widens as the educational stage progresses. The OR of PA compliance for females, compared to males in the primary school, middle school, and high school stages were 0.92, 0.88, and 0.83 respectively. Furthermore, during the high school stage, the odds of PA compliance amongst children and adolescents in the southern region were significantly lower than those in the northern region (OR = 0.88 95% CI:0.85–0.89; refer to Table 3).

### Recreational screen time

The analysis of compliance rate, trends for RST compliance rates by genders, areas, and regions, segregated by school levels are shown in sTable 2. In 2019, more than 80% of children and adolescents recorded less than 2 h of RST per day. Specifically, 80.76% (95% CI:77.93-83.58%) from primary schools, 82.86% (95% CI:78.99-86.72%) from middle schools, and 90.52% (95% CI:85.43-95.60%) from high schools (refer to Table 2).

**Table 3:** Weighted logistic regression models of physical activity and recreational screen time compliance among Chinese children and adolescents from 2017–2019 ^a^

	Odds Ratio (95% CI) ^b^					
	Physical Activity			Recreational Screen Time		
	Primary	Middle	High	Primary	Middle	High
**Sample Size**	22,143	13,320	5687	22,143	13,320	5687
**Grade** ^**c**^	0.98 (0.97 ,0.99)	1.01 (1.0 ,1.02)	1.13 (1.11 ,1.14)	1.0 (1.0 ,1.01)	1.01 (1.0 ,1.02)	0.97 (0.96 ,0.98)
**Gender**						
Male	1 [Reference]	1 [Reference]	1 [Reference]	1 [Reference]	1 [Reference]	1 [Reference]
Female	0.92 (0.9 ,0.92)	0.88 (0.86 ,0.89)	0.83 (0.8 ,0.84)	1.06 (1.05 ,1.07)	1.06 (1.05 ,1.07)	1.01 (0.99 ,1.02)
**Area** ^**d**^						
Urban	1 [Reference]	1 [Reference]		1 [Reference]	1 [Reference]	
Rural	0.97 (0.95 ,1.0)	1.02 (0.99 ,1.05)		1.01 (0.99 ,1.03)	1.01 (0.98 ,1.04)	
**Region** ^**d**^						
North	1 [Reference]	1 [Reference]	1 [Reference]	1 [Reference]	1 [Reference]	1 [Reference]
South	0.99 (0.98 ,1.0)	1.01 (0.99 ,1.02)	0.88 (0.85 ,0.89)	0.98 (0.97 ,0.99)	0.96 (0.95 ,0.97)	1.02 (1.0 ,1.04)

^a^ Participant characteristics are outlined according to their school level: primary, middle, and high school students, which collectively represent the population of Chinese students enrolled in school.

^b^ For categorical variables, the odds ratios (ORs) indicate the expected change in odds for each category relative to the reference group.

^c^ The ORs in this row denote the expected change in odds resulting from a one-year increase in grade within the specific school level.

^d^ Definitions for area and region are provided in the footnotes of Table 1.

**Fig. 2:**
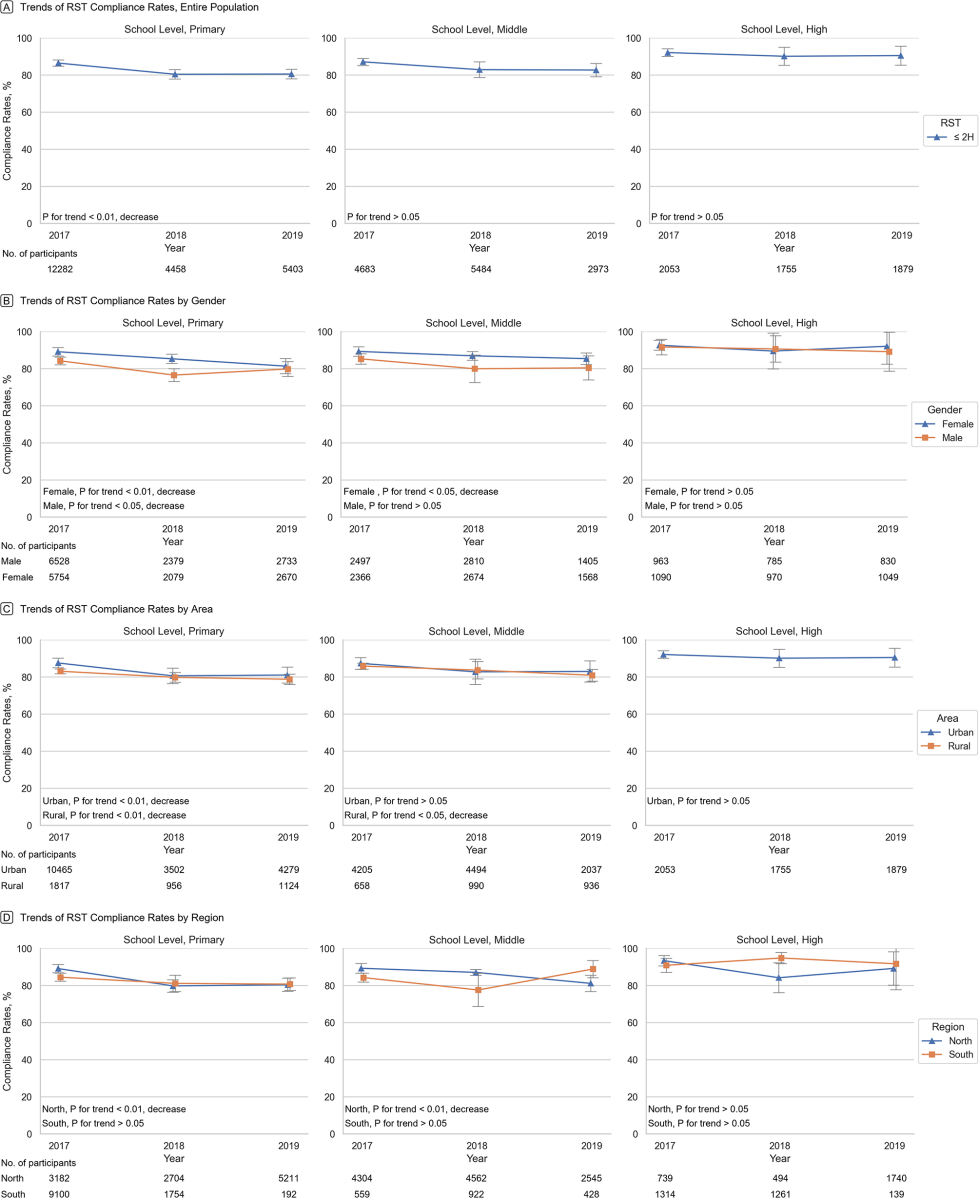
Crude weighted trends in recreational screen time from 20,017 − 2019. Data were weighted to be nationally representative. Error bars indicate 95% CIs

From 2017 to 2019, the average annual decrease in RST compliance rates was 3.18% (95% CI: 1.57-4.78%) for primary school students, and 4.17% (95% CI: 2.63-5.71%) for students in the northern regions. For male and female students, the average annual decrease in RST compliance rates was 2.42% (95% CI: 0.53-4.32%) and 3.08% (95% CI: 1.77-4.39%), respectively. For rural and urban students, the average annual decrease was 2.21% (95% CI: 1.04-3.38%) and 2.89% (95% CI: 1.16-4.62%), respectively. However, RST compliance rates remained constant for middle school and high school students, as well as for students from the southern region. The decline in RST compliance rates amongst primary school students was observed across genders, areas, and northern regions, but not for students from the southern regions. Although there was no significant overall decline in RST compliance rates amongst middle school students, there was a significant decline observed amongst middle school students that were female, from rural areas, and from northern regions between 2017 and 2019. Analysis of compliance rate trends for RST by genders, areas, and regions, categorized according to school levels can be found in sTable 2. For overall prevalence estimates, please refer to Table 2; Fig. 2.

When comparing the data collected between 2017 and 2019, we found that during the high school stage, RST compliance rates consistently decreases with each grade level increases (OR = 0.97, 95% CI:0.96–0.98 for each grade increment compared to the previous grade). We also found that across primary school and middle school stages, the RST compliance rates of females was significantly higher than males. The OR of RST compliance rates for females, compared to males, was 1.06 in primary and middle schools. Furthermore, during the primary school and middle school stages, the odds of RST compliance among children and adolescents in the southern region were significantly lower than those in the northern region, with ORs for primary and middle schools being 0.98 and 0.96 respectively (refer to Table 3).

## Discussion

This study explored the current trends in PA and RST among Chinese children and adolescents and examined how these trends vary by genders, school levels, areas (urban versus rural), and regions (north versus south) in China – a country that is experiencing rapid economic development and high levels of digitalization in Asia. Using 3 consecutive large-scale surveys conducted from 2017 to 2019, a significant decline in both PA and RST compliance rates amongst children and adolescents was observed. Notably, the decrease in PA compliance rate was mainly due to primary and middle school students, while the decline in RST compliance rate arose mainly from primary school students.

As emphasized in the policy guidelines from the “Physical Activity Guidelines for Americans”, understanding the trends of PA and RST is a crucial prerequisite for devising and implementing strategies to enhance PA and reduce RST [37]. However, according to the “Expert Consensus Statement on Physical Activity and Health in Chinese Children and Adolescents (2020)”, the unclear trends of PA and RST pose a myriad of challenges to launching effective health promotion initiatives aimed at increasing PA and reducing RST amongst Chinese children and adolescents [8]. Although global reviews [4, 5] and previous studies from Europe [38] and the United States [39] have identified the evolving trends and patterns of PA and RST amongst children and adolescents in their respective countries, these results are largely inconsistent. Additionally, results from previous studies lack precise depiction of the trends in PA and RST across different genders, school levels, regions, and areas in an Asian context. Furthermore, there is limited research examining the PA and RST trends of children and adolescents situated in highly digitalized countries with rapid economic development, such as China. China is characterized by gender-based expectations, academic pressure across different schooling stages, urban-rural dichotomy, a geographical range from tropics to temperate zones, along with swift socioeconomic changes since the 21st century. Hence, the present study offers substantial opportunities to bridge the research gap by providing a timely and contemporary understanding of current PA and RST trends amongst children and adolescents of different genders, school levels, areas, and regions from the lens of a rapid growing Asian country.

Contrary to previous studies that reported a gradual decline in the PA levels of children and adolescents with increasing grade levels [14, 40, 41], findings from the present study indicated that during the high school phase, students’ PA compliance rates actually increased with each advancing grade. This discrepancy may be attributed to the intense academic pressure endured by high school students in China – the National College Entrance Examination (Gaokao) taken by high school students is the solitary path to higher education and a brighter career path. Given that the number of students taking the national examination typically exceeds the total number of places offered by each university, the results of this two-day examination significantly influences students’ future academic trajectories [42]. As such, academic pressure increases significantly and high school students may participate in PA as a stress-relief outlet.

We also observed that students from the affluent southern region demonstrated lower levels of PA than their counterparts in the comparatively poorer northern regions. In the wealthier southern region, parents have more financial resources to support their children’s participation in various tuition classes to improve their examination results. This could explain the lower levels of PA participation amongst high school students in the southern region compared to their northern counterparts – more time is devoted to academics while reducing the amount of time available to engage in PA.

Our study also found a notable decline in PA compliance amongst primary and middle school students. This result contradicts the findings of Guthold et al. and Bucksch et al. which previously suggested that there was a slight increase in children and adolescents’ PA compliance in the early 20th century [4, 21]. However, Silva et al.’s study on Brazilian children and adolescents showed similar results to the present study – there was a significant downward trend in PA compliance in this age group [43]. As posited by Ng et al., the levels of PA is closely related to the economic development of a country [17]. Given that China and Brazil have undergone rapid economic development and social transformation during the past few decades, this could perhaps explain the rapid decline in PA amongst children and adolescents in these 2 countries. Interestingly, our study showed that the decline was more prominent in males (particularly in the primary schools) and not females unlike what was observed in Silvia et al.’s study on Brazilian children and adolescents. This difference in findings could be associated with social safety environments. In China, safety issues, such as theft or harassment, rarely affect females from engaging in PA, whereas in Brazil, safety is a major concern that hugely influences girls’ participation in outdoor PA [43]. 

Another interesting finding from our study is that in rural areas, the decline in PA compliance was most significant among primary school students, whereas in urban areas, it was the middle school students who experienced a more pronounced decrease in PA compliance. This could be attributed to academic pressure between primary and middle schools, as well as the urban-rural division. In China, unlike middle school and high school students who face pressures from important examinations such as the ‘Zhongkao’ (middle school graduation examination) or ‘Gaokao’ (college entrance examination), primary school students generally face less academic pressure. Thus, providing sufficient PA opportunities at this stage of their development may be able to curb the downward trend of their PA involvement. However, compared to urban parents who enroll their children in various extracurricular interest tutoring classes (such as basketball, martial arts, taekwondo) and take them to various PA venues (such as parks, gymnasiums etc.), rural areas in China often lag behind urban area in terms of availability of spaces, facilities, and opportunities for PA [44], resulting in the difference in PA habits among urban and rural primary school students. At middle schools, students start to feel the pressure of the high school entrance examination. This pressure tends to be more pronounced among urban students due to disparities in parental educational beliefs and levels of economic development between urban and rural areas. Compared to urban parents who invest more in academic tutoring for their children [45], rural parents often place less emphasis on the quality of their children’s education [46]. Some rural parents may even have their middle school-aged children assist with household or agricultural tasks. This might explain why rural middle school students have more opportunities for PA. This explains the difference in PA trends between urban and rural middle school students.

Another crucial finding from our study is the changing trends of PA amongst primary school students. We found that the downward trend is more pronounced in students residing in the northern compared to the south region. This could be due to the differences in economic development level and environmental factors between the 2 regions. In the past decade, economic development in southern China has surpassed that of the north, with statistical data from 2019 indicating that the GDP of the northern region accounted for only 35% of China’s total GDP [47]. As a result, the southern region has more fund and resources to invest in public sports facilities such as stadiums and parks, which could delay the decline in PA rates. Conversely, people in the northern region may lack these facilities to engage in PA. Furthermore, unfavorable weather conditions and increasing air pollution in the north may severely limit outdoor PA opportunities, leading to a significant decline in PA in this region [35]. 

In light of the key findings from the present study, as well as the impact of lower PA on the physical and mental health of children and adolescents [3], future interventions should aim to mitigate the negative impacts of economic development by directing more economic growth income towards the construction of public sports facilities. Consequently, this provides more opportunities for the public to engage in outdoor PA. The formulation and implementation of fun and engaging programs to encourage more Chinese children and adolescents to increase their PA levels in and outside school is also imperative. One example is to gather feedback from the students on the types of new sports and games that excite them, and incorporate them in the physical education curriculum. Future policies could also aim to address and reduce the amount of academic pressure placed on middle and high school students, for example, by requiring schools to reduce the assignment of homework and limiting the establishment of academic tutoring classes may provide more times children and adolescents to engage in PA.

Our result indicates that the RST compliance among children and adolescents in the southern region is significantly lower than of the northern region. This disparity might stem from the varying economic conditions across northern and southern regions of China. From 2008 to 2019, southern China experienced a per capita GDP growth rate of 9.42%, outpaced northern China with a growth rate of 8.32% [47]. This accelerated economic development in the south has resulted in a higher proliferation of electronic devices (such as computers, tablets, or mobile phones) among children and adolescents. Consequently, contributing to a decrease in RST compliance in the southern region.

Our study’s results also indicated a substantial decrease in RST compliance among primary school children and adolescents from 2017 to 2019. This aligns with global trends, which shown a significant worldwide increase in RST during the first 20 years of the 21st century [20, 48]. The primary cause of this trend is the rapid proliferation of televisions, computers, mobile phones, and tablets since the beginning of the 21st century [9, 10]. Interestingly, the drop in RST compliance was not significant among primary and middle school students in the southern region, but it was significant in the north, rural area, and among female middle school students. We attribute this trend to the increased availability of electronic devices in the northern and rural regions during 2017–2019 [12]. In the more affluent southern and urban regions, most students already had the necessary electronic devices before 2017 [12], which could explain the insignificant changes in their RST compliance rate. Furthermore, the significant decrease in RST compliance amongst female middle school students may be attributed to lifestyle changes associated with puberty. As Hardy suggested, interpersonal issues and socializing become central aspects of girls’ lives as they enter adolescence, leading them to allocate more time for online chatting with friends, which contributed to an increased in their RST [49]. 

Given the detrimental effects of extended RST on the physical and mental health of children and adolescents [48], findings from the present study suggest that the government should consider implementing policies to mitigate children and adolescents’ electronic device usage time. Schools should offer interesting PA programs (e.g. orienteering, hip hop dancing etc.) for female middle school students beyond running or walking with their friends to enhance their motivation in PA participation.

To the best of our knowledge, this is the first study to explore current trends in PA and RST amongst children and adolescents of different genders, school levels, areas, and regions in a rapid development and digitalized country. The strengths of this study include the use of a national representative sample, consistent data collection over 3 consecutive years using the same instruments, and a detailed analysis of varying trends in PA and RST amongst children and adolescents.

Although this study makes notable contributions to the literature, there are several limitations that are worth noting. First, the self-report survey tools may not accurately represent the actual PA and RST of children and adolescents. Therefore, there may be slight deviations in the calculation of compliance rates for PA and RST. Although these errors do not affect our estimation of the trend changes in PA and RST for children and adolescents, future studies should employ objective measures such as triaxial accelerometer to enhance the study design. Second, the PA questionnaire does not provide information regarding the duration and frequency of PA. Therefore, in this study, the calculation of PA compliance is based on the research results of Voss rather than the guidelines from WHO. This reduces the comparability of this study with other studies. Future research should consider tools that include intensity, frequency, and duration of PA. Lastly, our study solely focused on RST, and did not include time spent using computers or other electronic devices for educational purposes. Our study also did not consider other types of sedentary behavior, such as educational, social, and transportation time. Future research should examine the different types of SB to obtain a more accurate distribution of sedentary time amongst children and adolescents.

## Conclusions

The present study found significant decline in PA compliance rates of primary and middle school students, and in RST compliance rates of primary school students in China from 2017 to 2019. This downward trend varies by school levels, genders, areas, and regions. From a public health and health education perspective, this study underscores the urgency of increasing PA and reducing RST among middle school and primary school students. Future policies and research should pay closer attention to the impact of rapid economic development and digitization on the PA and RST behaviors of children and adolescents, and provide specific and targeted programs to promote the development of an active and healthy youthful citizen.

### Electronic supplementary material

Below is the link to the electronic supplementary material.

Supplementary Material 1

## Data Availability

The datasets analyzed in the current study are available from the corresponding author on reasonable request.

## References

[1] Caspersen CJ, Powell KE, Christenson GM (1985). Physical activity, exercise, and physical fitness: definitions and distinctions for health-related research. Public Health Rep.

[2] Tremblay MS, Carson V, Chaput J-P (2016). Canadian 24-Hour Movement Guidelines for Children and Youth: an integration of physical activity, sedentary Behaviour, and Sleep. Appl Physiol Nutr Metab.

[3] World Health Organization. WHO guidelines on physical activity and sedentary behaviour. https://www.who.int/publications-detail-redirect/9789240015128. [accessed 16.10.2022]. 33369898

[4] Guthold R, Stevens GA, Riley LM (2020). Global trends in insufficient physical activity among adolescents: a pooled analysis of 298 population-based surveys with 1·6 million participants. Lancet Child Adolesc Health.

[5] Aubert S, Barnes JD, Demchenko I (2022). Global matrix 4.0 physical activity report card grades for children and adolescents: results and analyses from 57 countries. J Phys Activity Health.

[6] Aubert S, Barnes J, Abdeta C (2018). Global matrix 3.0 physical activity report card grades for children and youth: results and analysis from 49 countries. J Phys Activity Health.

[7] Li F, Mao L, Chen P (2019). Physical activity and prevention of chronic disease in Chinese youth: a public health approach. J Sport Health Sci.

[8] Chen P, Wang D, Shen H (2020). Physical activity and health in Chinese children and adolescents: expert consensus statement (2020). Br J Sports Med.

[9] Cui Z, Hardy LL, Dibley MJ (2011). Temporal trends and recent correlates in sedentary behaviours in Chinese children. Int J Behav Nutr Phys Act.

[10] Zhang J, Seo D-C, Kolbe L (2012). Associated trends in sedentary behavior and BMI among Chinese school children and adolescents in seven diverse Chinese provinces. IntJ Behav Med.

[11] National Bureau of Statistics (2020). China Statistical Yearbook 2020.

[12] Office of the Central Cyberspace Affairs Commission of the Communist Party of China. The 45th Statistical Report on the Development of China’s Internet. http://www.cac.gov.cn/2020-04/27/c_1589535470378587.htm. [accessed 27.04.2023].

[13] Brodersen NH, Steptoe A, Boniface DR (2007). Trends in physical activity and sedentary behaviour in adolescence: ethnic and socioeconomic differences. Br J Sports Med.

[14] Obradovich N, Fowler JH (2017). Climate change may alter human physical activity patterns. Nat Hum Behav.

[15] Bernard P, Chevance G, Kingsbury C (2021). Climate change, physical activity, and sport: a systematic review. Sports Med.

[16] Dallolio L, Marini S, Masini A (2022). The impact of COVID-19 on physical activity behaviour in Italian primary school children: a comparison before and during pandemic considering gender differences. BMC Public Health.

[17] Ng SW (2012). Time use and physical activity: a shift away from movement across the globe. Obes Rev.

[18] Knuth AG, Hallal PC (2009). Temporal trends in physical activity: a systematic review. J Phys Act Health.

[19] Bassett DR, John D, Conger SA (2015). Trends in physical activity and sedentary behaviors of United States youth. J Phys Activity Health.

[20] Yang L, Cao C, Kantor ED (2019). Trends in Sedentary Behavior among the US Population, 2001–2016. JAMA.

[21] Kalman M, Inchley J, Sigmundová D (2015). Secular trends in moderate-to-vigorous physical activity in 32 countries from 2002 to 2010: a cross-national perspective. Eur J Public Health.

[22] Tremblay MS, Barnes JD, González SA (2016). Global matrix 2.0: report card grades on the physical activity of children and youth comparing 38 countries. J Phys Activity Health.

[23] QuestionStar. QuestionStar, more than just surveys and online exams. https://www.wjx.cn. [ac-cessed 02.05.2023]. [in Chinese].

[24] Baid. Baidu Map Open Platform. https://lbsyun.baidu.com/. [accessed 02.05.2023]. [in Chinese].

[25] National Bureau of Statistics (2018). China Statistical Yearbook 2018.

[26] National Bureau of Statistics (2019). China Statistical Yearbook 2019.

[27] Tukey JW (1977). Exploratory data analysis.

[28] Kowalski KC, Crocker PR, Donen RM et al. The physical activity questionnaire for older children (PAQ-C) and adolescents (PAQ-A) manual. 2004.

[29] Voss C, Dean PH, Gardner RF (2017). Validity and reliability of the physical activity questionnaire for children (PAQ-C) and adolescents (PAQ-A) in individuals with congenital heart disease. PLoS ONE.

[30] Wang JJ, Baranowski T, Lau WP (2016). Validation of the physical activity questionnaire for older children (PAQ-C) among Chinese children. Biomed Environ Sci.

[31] Hardy LL, Booth ML, Okely AD (2007). The reliability of the adolescent sedentary activity questionnaire (ASAQ). Prev Med.

[32] Qiang G. A study on physical activity levels among Chinese children and adolescents and their influencing factors. East China Normal University; 2016. [in Chinese].

[33] Zhang Y, Ma S, Chen C, Liu S, Zhang C, Cao Z et al. Physical activity guidelines for children and adolescents in China. Chin J Evidence-Based Pediatr, 12, 401–9. [in Chinese].

[34] Ministry of Education. Further improving the enrollment process for primary and secondary schools. Availa-ble at: http://www.moe.gov.cn/srcsite/A06/s3321/202204/t20220401_612689.html. [accessed 02.05.2023]. [in Chinese].

[35] Liu G (2010). Atlas of Natural Geography of China.

[36] Python Organization, Python. https://www.python.org/. [accessed 28.01.2023].

[37] U.S. Department of Health and Human Services. Physical Activity Guidelines for Americans 2 edition. https://health.gov/paguidelines/second-edition/pdf/Physical_Activity_Guidelines_2nd_edition.pdf. [accessed 08.06.2022].

[38] Arnarsson (2020). Spotlight on adolescent health and well-being: findings from the 2017/2018 Health Behaviour in School-aged children (HBSC) survey in Europe and Canada International report.

[39] Katzmarzyk PT, Denstel KD, Beals K (2018). Results from the United States 2018 report card on physical activity for children and youth. J Phys Activity Health.

[40] Dumith SC, Gigante DP, Domingues MR (2011). Physical activity change during adolescence: a systematic review and a pooled analysis. Int J Epidemiol.

[41] Fan X, Cao Z-B (2017). Physical activity among Chinese school-aged children: National prevalence estimates from the 2016 physical activity and fitness in China—the Youth Study. J Sport Health Sci.

[42] Cai X, Lu Y, Pan J (2019). Gender gap under pressure: evidence from China’s national college entrance examination. Rev Econ Stat.

[43] Pinto AA, Fernandes RA, da Silva KS et al. Physical Activity Levels in Brazilian Adolescents: A Secular Trend Study (2007–2017/18). IJERPH. 2022; 19: 16901. 10.3390/ijerph192416901. 10.3390/ijerph192416901 PMC9779524 36554783

[44] Lu C, Stolk RP, Sauer PJJ (2017). Factors of physical activity among Chinese children and adolescents: a systematic review. Int J Behav Nutr Phys Act.

[45] Dai K (2023). Double reduction policy in education industry and firm values: evidence from China. Finance Res Lett.

[46] Zheng X, Wang C, Shen Z (2020). Associations of private tutoring with Chinese students’ academic achievement, emotional well-being, and parent-child relationship. Child Youth Serv Rev.

[47] Xie D, Bai C, Xiao W (2022). Institutional environment, development model transformation and north–south economic disparity in China. Growth Change.

[48] Patterson R, McNamara E, Tainio M (2018). Sedentary behaviour and risk of all-cause, cardiovascular and cancer mortality, and incident type 2 diabetes: a systematic review and dose response meta-analysis. Eur J Epidemiol.

[49] Hardy LL, Bass SL, Booth ML (2007). Changes in sedentary behavior among adolescent girls: a 2.5-year prospective cohort study. J Adolesc Health.

