# Supplementary material for: Trends of physical activity and recreational screen time among Chinese children and adolescents: a national study from 2017 to 2019
Source: BMC Public Health. 2024 May 13;24:1305. doi: 10.1186/s12889-024-18822-1 (PMC11092243; doi:10.1186/s12889-024-18822-1)
Supplement: Supplementary file 1 — Supplementary Material 1 [file 12889_2024_18822_MOESM1_ESM.docx]

**Supplementary Materials**

**sTable** **1** Weighted sample size for physical activity and recreational screen time in Chinese children and adolescents 2017-2019 ^a^.

|  | No. of Participants by Grade Level (Weight %) | | | | | | | | |
| --- | --- | --- | --- | --- | --- | --- | --- | --- | --- |
|  | 2017 | | | 2018 | | | 2019 | | |
|  | Elementary | Middle | High | Elementary | Middle | High | Elementary | Middle | High |
| **Overall** | 100936918 | 44420568 | 23745453 | 103392479 | 46525792 | 23753678 | 105612296 | 48271300 | 24143019 |
| **Gender** |  |  |  |  |  |  |  |  |  |
| Male | 54745085 | 24086901 | 12837961 | 55968194 | 25229984 | 12880189 | 57162079 | 26249728 | 13087311 |
| Female | 46191833 | 20333667 | 10907492 | 47424285 | 21295808 | 10873489 | 48450217 | 22021572 | 11055708 |
| **Area^b^** |  |  |  |  |  |  |  |  |  |
| Urban | 73183323 | 37986505 | 23745453 | 76728372 | 40041761 | 23753678 | 80037252 | 41767096 | 24143019 |
| Rural | 27753595 | 6434063 | 0^e^ | 26664107 | 6484031 | 0^e^ | 25575044 | 6504204 | 0^e^ |
| **Region^c^** |  |  |  |  |  |  |  |  |  |
| North | 40985315 | 18318882 | 10316590 | 41856290 | 19242385 | 10216647 | 42764133 | 19913815 | 10276058 |
| South | 59951603 | 26101686 | 13428863 | 61536189 | 27283407 | 13537031 | 62848163 | 28357485 | 13866961 |

^a^ Participant characteristics were presented according to year and school level: primary school students, middle school students, high school students.

^b^ Area classifications were determined based on the urban-rural designation of each school, as provided by the Baidu Maps Open Platform.

^c^ Regions were defined according to the natural geographic boundaries of China, specifically the Qinling-Huaihe Line.

^e^ Due to the predominant placement of Chinese high schools in urban areas, the value in this context is 0.

**sTable 2** Crude weighted trends in physical activity and recreational screen time compliance rates among Chinese children and adolescents from 2017-2019^a^.

|  | Trends in Physical Activity and Recreational Screen Time Compliance Rates | | | | | |
| --- | --- | --- | --- | --- | --- | --- |
|  | 2017 | 2018 | 2019 | β (95% CI) ^b^ | *P* for Trend^b^ | 2019 VS 2017 Difference (95% CI)^c^ |
| **Primary School PA Compliance Rates, Weighted % (95% CI)** | | | | | | |
| Gender |  |  |  |  |  |  |
| Male | 37.28 (33.11, 41.46) | 34.99 (29.34, 40.64) | 27.77 (21.17, 34.36) | -4.58 (-8.17, -0.99) | 0.014 | 37.28 (33.11, 41.46) |
| Female | 27.07 (22.83, 31.31) | 27.98 (22.62, 33.33) | 22.46 (16.27, 28.64) | -2.09 (-5.53, 1.35) | 0.228 | 27.07 (22.83, 31.31) |
| Area |  |  |  |  |  |  |
| Rural | 34.51 (31.37, 37.64) | 32.7 (28.99, 36.42) | 28.76 (25.19, 32.34) | -2.78 (-5.17, -0.4) | 0.023 | 34.51 (31.37, 37.64) |
| Urban | 31.99 (26.8, 37.19) | 31.64 (25.28, 38.0) | 24.31 (17.09, 31.53) | -3.61 (-7.76, 0.54) | 0.087 | 31.99 (26.8, 37.19) |
| Region |  |  |  |  |  |  |
| North | 36.35 (30.53, 42.18) | 28.88 (24.41, 33.34) | 26.58 (20.96, 32.19) | -4.86 (-8.44, -1.29) | <0.01 | 36.35 (30.53, 42.18) |
| South | 29.88 (26.06, 33.7) | 35.39 (28.59, 42.19) | 22.82 (14.57, 31.08) | -1.97 (-6.2, 2.25) | 0.352 | 29.88 (26.06, 33.7) |
| **Middle School PA Compliance Rates, Weighted % (95% CI)** | | | | | | |
| Gender |  |  |  |  |  |  |
| Male | 33.69 (28.08, 39.31) | 32.72 (29.21, 36.23) | 21.1 (17.37, 24.82) | -6.23 (-9.3, -3.16) | <0.01 | -12.6 (-21.23, 1.0) |
| Female | 21.59 (17.33, 25.84) | 15.7 (12.12, 19.27) | 13.46 (9.52, 17.4) | -4.09 (-6.67, -1.51) | <0.01 | -8.13 (-12.89, 5.62) |
| Area |  |  |  |  |  |  |
| Rural | 32.16 (25.53, 38.79) | 28.43 (23.24, 33.63) | 26.29 (22.75, 29.83) | -2.95 (-6.32, 0.42) | 0.084 | -5.86 (-13.78, 6.07) |
| Urban | 27.46 (21.17, 33.74) | 25.1 (18.02, 32.19) | 16.2 (12.22, 20.19) | -5.59 (-9.47, -1.7) | <0.01 | -11.25 (-21.02, 1.24) |
| Region |  |  |  |  |  |  |
| North | 25.88 (21.1, 30.65) | 25.25 (20.27, 30.23) | 18.02 (14.23, 21.82) | -4.07 (-6.99, -1.15) | <0.01 | -7.85 (-12.49, 4.6) |
| South | 31.09 (23.44, 38.74) | 25.88 (16.74, 35.02) | 15.96 (11.74, 20.18) | -7.11 (-13.05, -1.17) | 0.021 | -15.13 (-24.55, 3.83) |
| **High School PA Compliance Rates, Weighted % (95% CI)** | | | | | | |
| Gender |  |  |  |  |  |  |
| Male | 35.11 (19.71, 50.51) | 41.79 (10.08, 73.49) | 17.77 (0.27, 35.26) | -7.38 (-23.05, 8.28) | 0.329 | -17.34 (-42.36, 18.65) |
| Female | 10.04 (4.7, 15.39) | 26.9 (-4.84, 58.63) | 6.4 (1.98, 10.82) | -0.24 (-14.95, 14.47) | 0.973 | -3.65 (-16.97, 7.03) |
| Area |  |  |  |  |  |  |
| Rural |  |  |  |  |  |  |
| Urban | 23.59 (12.83, 34.36) | 34.97 (16.01, 53.93) | 12.56 (4.37, 20.75) | -4.09 (-14.98, 6.79) | 0.448 | -11.03 (-27.21, 10.39) |
| Region |  |  |  |  |  |  |
| North | 29.11 (8.87, 49.34) | 55.45 (19.06, 91.84) | 17.61 (5.1, 30.12) | -3.28 (-21.89, 15.34) | 0.714 | -11.5 (-39.97, 18.98) |
| South | 18.45 (3.72, 33.18) | 18.6 (9.32, 27.89) | 7.71 (-13.66, 29.09) | -4.87 (-12.9, 3.17) | 0.212 | -10.74 (-47.4, 25.73) |
| **Primary School RST Compliance Rates, Weighted % (95% CI)** | | | | | | |
| Gender |  |  |  |  |  |  |
| Male | 84.22 (82.11, 86.33) | 76.55 (73.12, 79.99) | 79.84 (75.84, 83.85) | -2.58 (-4.84, -0.32) | 0.026 | -4.38 (-7.59, 2.54) |
| Female | 89.14 (86.8, 91.48) | 85.35 (82.87, 87.83) | 81.38 (77.32, 85.45) | -3.87 (-5.72, -2.02) | <0.01 | -7.75 (-10.6, 0.78) |
| Area |  |  |  |  |  |  |
| Rural | 83.1 (81.79, 84.41) | 79.82 (77.17, 82.47) | 78.8 (76.03, 81.56) | -2.25 (-3.7, -0.8) | <0.01 | -4.31 (-5.77, 1.81) |
| Urban | 87.57 (85.0, 90.14) | 80.64 (76.47, 84.8) | 81.07 (76.76, 85.38) | -3.49 (-5.97, -1.02) | <0.01 | -6.5 (-12.07, 1.17) |
| Region |  |  |  |  |  |  |
| North | 89.17 (86.94, 91.39) | 79.83 (76.47, 83.19) | 80.43 (76.87, 84.0) | -4.32 (-6.6, -2.04) | <0.01 | -8.73 (-10.84, -0.57) |
| South | 84.5 (82.32, 86.69) | 81.17 (76.76, 85.57) | 80.78 (77.46, 84.1) | -2.12 (-4.5, 0.27) | 0.081 | -3.72 (-8.62, 1.89) |
| **Middle School RST Compliance Rates, Weighted % (95% CI)** | | | | | | |
| Gender |  |  |  |  |  |  |
| Male | 85.3 (82.5, 88.1) | 79.96 (72.56, 87.37) | 80.45 (73.93, 86.98) | -2.46 (-6.5, 1.57) | 0.224 | -4.85 (-9.19, 6.88) |
| Female | 89.31 (86.72, 91.9) | 86.95 (84.66, 89.25) | 85.43 (82.31, 88.56) | -1.94 (-3.71, -0.17) | 0.032 | -3.88 (-6.72, 4.24) |
| Area |  |  |  |  |  |  |
| Rural | 85.9 (84.27, 87.53) | 83.71 (79.02, 88.41) | 80.96 (77.77, 84.15) | -2.46 (-4.58, -0.35) | 0.024 | -4.94 (-6.48, 2.66) |
| Urban | 87.35 (84.18, 90.52) | 82.8 (75.97, 89.63) | 83.01 (77.28, 88.74) | -2.2 (-5.81, 1.41) | 0.225 | -4.34 (-9.12, 8.16) |
| Region |  |  |  |  |  |  |
| North | 89.33 (86.72, 91.95) | 87.1 (85.49, 88.71) | 81.19 (76.76, 85.62) | -4.15 (-6.56, -1.75) | <0.01 | -8.14 (-10.37, 1.41) |
| South | 84.3 (81.91, 86.68) | 77.62 (68.67, 86.58) | 88.92 (84.3, 93.55) | 0.58 (-4.5, 5.65) | 0.818 | 4.62 (-4.14, 9.91) |
| **High School RST Compliance Rates, Weighted % (95% CI)** | | | | | | |
| Gender |  |  |  |  |  |  |
| Male | 91.7 (87.51, 95.9) | 90.67 (83.54, 97.79) | 89.18 (78.71, 99.66) | -1.24 (-5.17, 2.69) | 0.509 | -2.52 (-21.4, 14.44) |
| Female | 92.65 (89.98, 95.32) | 89.54 (79.86, 99.21) | 92.1 (82.44, 101.75) | -0.52 (-5.13, 4.09) | 0.813 | -0.56 (-16.89, 13.29) |
| Area |  |  |  |  |  |  |
| Rural |  |  |  |  |  |  |
| Urban | 92.14 (90.05, 94.23) | 90.15 (85.29, 95.01) | 90.52 (85.43, 95.6) | -0.91 (-3.68, 1.86) | 0.508 | -1.62 (-11.28, 6.0) |
| Region |  |  |  |  |  |  |
| North | 93.44 (90.64, 96.24) | 84.23 (76.18, 92.28) | 89.23 (80.21, 98.26) | -2.65 (-7.32, 2.02) | 0.246 | -4.2 (-17.28, 7.08) |
| South | 90.93 (87.2, 94.66) | 94.88 (91.88, 97.88) | 91.75 (77.82, 105.68) | 0.73 (-1.87, 3.34) | 0.551 | 0.82 (-16.72, 18.89) |

^a^ Weighted estimates and 95% confidence intervals (CIs) were calculated for each survey year. These estimates were adjusted to provide nationally representative data.

^b^ The parameter estimates β, corresponding 95% CIs, and P values for trend were obtained using linear regression, treating the year cycle as a continuous variable. β can be interpreted as the average percentage point change in prevalence per annum.

^c^ A value below zero indicates a decrease, corresponding to a negative difference.
